# *trans*-Dibromidobis(3-methyl­pyridine-κ*N*)copper(II)

**DOI:** 10.1107/S1600536813001414

**Published:** 2013-01-19

**Authors:** Firas F. Awwadi

**Affiliations:** a Department of Chemistry, The University of Jordan, Amman 11942, Jordan

## Abstract

The asymmetric unit of the title compound, [CuBr_2_(C_6_H_7_N)_2_], contains one half-mol­ecule, the whole mol­ecule being generated by inversion through a center located at the Cu^II^ atom. The geometry around the Cu^II^ atom is square planar. Semicoordinate Cu⋯Br bonds [3.269 (1) Å] and nonclassical C—H⋯Br hydrogen bonds connect the mol­ecules, forming chains running parallel to the *a* axis. These chains are further linked *via* additional C—H⋯Br hydrogen bonds into a three-dimensional network.

## Related literature

The title compound was prepared to investigate chloro-methyl and bromo-methyl exchange rules in the crystal structures of [Cu(3YP)_2_Br_2_] complexes (where 3*Y*P = 3-substituted pyridine and *Y* = Cl, Br and meth­yl), see: Awwadi *et al.* (2006 ▶, 2011 ▶). Desiraju showed that the chloro-methyl exchange rule is obeyed if the final structure is stabilized by dispersive forces, see: Desiraju & Sarma (1986 ▶). For related structures, see: Marsh *et al.* (1981 ▶, 1982 ▶); Singh *et al.* (1972 ▶).
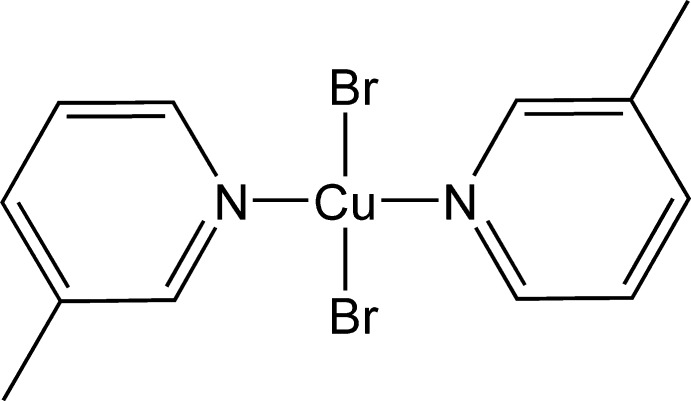


## Experimental

### 

#### Crystal data

[CuBr_2_(C_6_H_7_N)_2_]
*M* *_r_* = 409.61
Monoclinic, 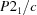

*a* = 4.0171 (8) Å
*b* = 14.105 (3) Å
*c* = 11.899 (2) Å
β = 92.54 (3)°
*V* = 673.5 (2) Å^3^
*Z* = 2
Mo *K*α radiation
μ = 7.53 mm^−1^
*T* = 85 K
0.24 × 0.03 × 0.03 mm

#### Data collection

Bruker/Siemens SMART APEX diffractometer
Absorption correction: multi-scan (*SADABS*; Bruker, 2001 ▶) *T*_min_ = 0.265, *T*_max_ = 0.806
5995 measured reflections
1536 independent reflections
1283 reflections with *I* > 2σ(*I*)
*R*_int_ = 0.044

#### Refinement

*R*[*F* ^2^ > 2σ(*F* ^2^)] = 0.032
*wR*(*F* ^2^) = 0.075
*S* = 1.01
1536 reflections
80 parameters
H-atom parameters constrained
Δρ_max_ = 0.97 e Å^−3^
Δρ_min_ = −0.47 e Å^−3^

### 

Data collection: *SMART* (Bruker, 2002 ▶); cell refinement: *SAINT-Plus* (Bruker, 2001 ▶); data reduction: *SAINT-Plus*; program(s) used to solve structure: *SHELXTL* (Sheldrick, 2008 ▶); program(s) used to refine structure: *SHELXTL*; molecular graphics: *SHELXTL*; software used to prepare material for publication: *SHELXTL*.

## Supplementary Material

Crystal structure: contains datablock(s) I, global. DOI: 10.1107/S1600536813001414/lr2097sup1.cif

Structure factors: contains datablock(s) I. DOI: 10.1107/S1600536813001414/lr2097Isup2.hkl

Additional supplementary materials:  crystallographic information; 3D view; checkCIF report

## Figures and Tables

**Table 1:** Hydrogen-bond geometry (Å, °)

*D*—H⋯*A*	*D*—H	H⋯*A*	*D*⋯*A*	*D*—H⋯*A*
C2—H2⋯Br1^i^	0.95	2.83	3.549 (4)	133
C6—H6⋯Br1^ii^	0.95	2.79	3.529 (4)	135
C5—H5⋯Br1^iii^	0.95	2.99	3.668 (4)	130

Symmetry codes: (i) 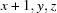; (ii) 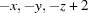; (iii) 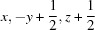.

**Fig. 1.**
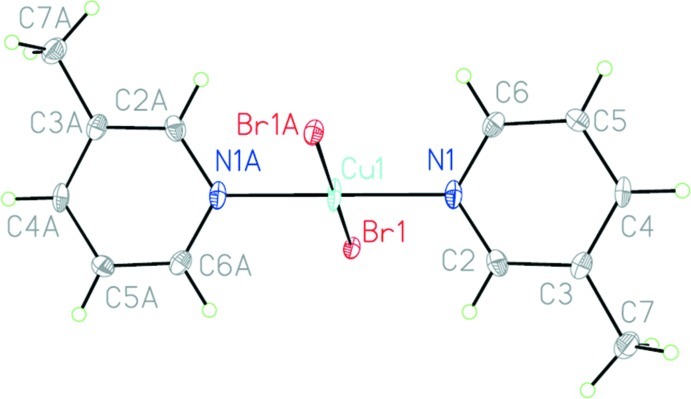
The molecular unit of the title compound. Symmetry transformations used to generate equivalent atoms are -x + 1, -y, -z + 2. Thermal ellipsoids are shown at 50% probability.

**Fig. 2.**
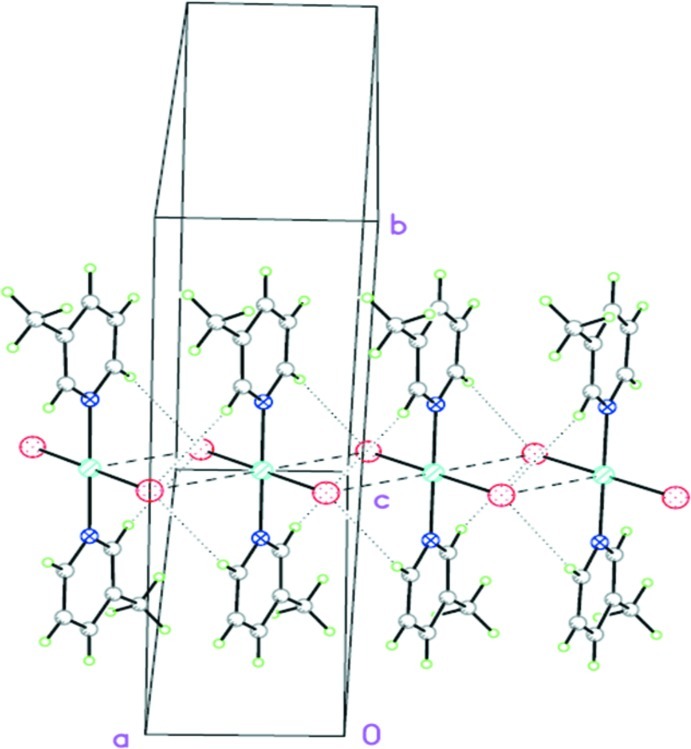
Chain structure of the title compound.

**Fig. 3.**
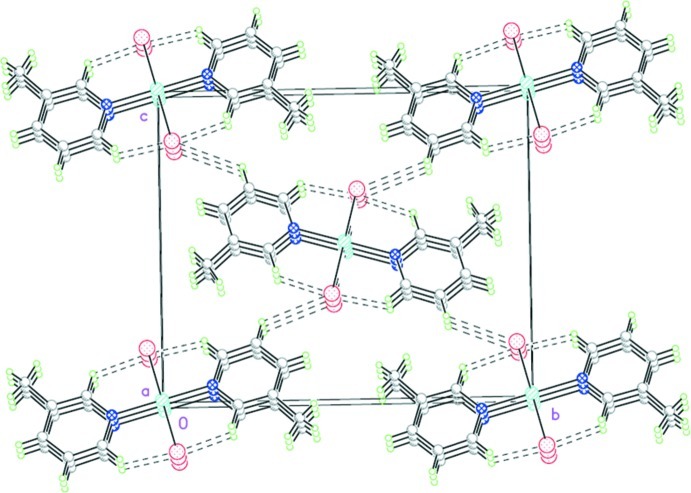
The packing diagram of the title compound viewed down the a-axis.

## supplementary crystallographic information

### Comment

The molecular units (Fig. 1) of the title compound are linked *via* Cu···Br semi-coordinate bonds to form a chain structure that runs parallel to the *a*-axis (Fig. 2). These chains are reinforced by C6—H6···Br1 and C2—H2···Br1 hydrogen bonding interactions. The data summarizing these interactions are shown in Table 1. These chains are interlinked using non-classical C5—H5···Br1 hydrogen bonding interactions to form the final three dimensional structure (Fig. 3).

Cu(4MP)_2_Cl_2_, (Marsh *et al.*, 1981), where 4MP is 4-methylpyridine, forms an extended chain structure based on the Cu···Cl semi coordinate bond, similar to the title compound. In contrast, Cu(2MP)_2_*X*_2_, 2MP = 2-methylpyridine and *X* = Cl or Br, form a dimer structure based on the Cu···*X* semi coordinate bond (Singh *et al.*, 1972 and Marsh *et al.*, 1982).

The title compound was prepared to investigate chloro-methyl and bromo-methyl exchange rules in the crystal structures of Cu(3YP)_2_Br_2_ complexes, where 3YP = 3-substituted pyridine and Y = Cl, Br and methyl (Awwadi *et al.*, 2006 and Awwadi *et al.*, 2011). These three compounds are isostructural in the solid state, hence, the halo-methyl exchange rule is not violated. Desiraju showed that the chloro-methyl exchange rule is obeyed if the final structure is stabilized by dispersive forces (Desiraju & Sarma, 1986). This indicates that the Cu···Br semi-coordinate bonds play the crucial role in determining the final structure of these compounds. The volume of the methyl group is *ca* 24 Å^3^ which is in between the volume of chlorine (*ca* 19 Å^3^) and bromine (*ca* 27 Å^3^). In contrast, if directional forces are involved, the chloro-methyl exchange rule is violated.

### Experimental

2 mmol of 3-methylpyridine were dissolved in 20 mL of acetonitrile. One mmol of CuBr_2_ was dissolved in 20 mL of acetonitrile. The two solutions were mixed. The resulting solution was gently heated with stirring for 15 minutes. The solution was filtered and left to slowly evaporate at the room temperature. Green crystals with a needle habit were formed. One of these crystals was used for single-crystal X-ray data collection.

### Refinement

The structure was solved by direct methods and refined by least squares method on F^2^ using the *SHELXTL* program package. The structure was solved in the space group P2(1)/c (# 14) by analysis of systematic absences. All atoms were refined anisotropically. Hydrogen atoms were placed at the calculated positions using a riding model with C(aromatic)—H = 0.95 Å and *Uiso*(H) = 1.2*Ueq*(C), and with C(aliphatic)—H = 0.98 Å and *Uiso*(H) = 1.5*Ueq*(C).

### Crystal data

**Table:**

[CuBr_2_(C_6_H_7_N)_2_]	*F*(000) = 398
*M**_r_* = 409.61	*D*_x_ = 2.020 Mg m^−^^3^
Monoclinic, *P*2_1_/*c*	Mo *K*α radiation, λ = 0.71073 Å
Hall symbol: -P 2ybc	Cell parameters from 2369 reflections
*a* = 4.0171 (8) Å	θ = 2.2–29.8°
*b* = 14.105 (3) Å	µ = 7.53 mm^−^^1^
*c* = 11.899 (2) Å	*T* = 85 K
β = 92.54 (3)°	Needle, green
*V* = 673.5 (2) Å^3^	0.24 × 0.03 × 0.03 mm
*Z* = 2	

### Data collection

**Table:**

Bruker/Siemens SMART APEX diffractometer	1536 independent reflections
Radiation source: normal-focus sealed tube	1283 reflections with *I* > 2σ(*I*)
Graphite monochromator	*R*_int_ = 0.044
Detector resolution: 8.3 pixels mm^-1^	θ_max_ = 27.5°, θ_min_ = 2.9°
ω scans	*h* = −5→4
Absorption correction: multi-scan (*SADABS*; Bruker, 2001)	*k* = −16→18
*T*_min_ = 0.265, *T*_max_ = 0.806	*l* = −14→15
5995 measured reflections	

### Refinement

**Table:**

Refinement on *F*^2^	Primary atom site location: structure-invariant direct methods
Least-squares matrix: full	Secondary atom site location: difference Fourier map
*R*[*F*^2^ > 2σ(*F*^2^)] = 0.032	Hydrogen site location: inferred from neighbouring sites
*wR*(*F*^2^) = 0.075	H-atom parameters constrained
*S* = 1.01	*w* = 1/[σ^2^(*F*_o_^2^) + (0.0388*P*)^2^] where *P* = (*F*_o_^2^ + 2*F*_c_^2^)/3
1536 reflections	(Δ/σ)_max_ < 0.001
80 parameters	Δρ_max_ = 0.97 e Å^−^^3^
0 restraints	Δρ_min_ = −0.47 e Å^−^^3^

### Special details

**Table:**

Geometry. All e.s.d.'s (except the e.s.d. in the dihedral angle between two l.s. planes) are estimated using the full covariance matrix. The cell e.s.d.'s are taken into account individually in the estimation of e.s.d.'s in distances, angles and torsion angles; correlations between e.s.d.'s in cell parameters are only used when they are defined by crystal symmetry. An approximate (isotropic) treatment of cell e.s.d.'s is used for estimating e.s.d.'s involving l.s. planes.
Refinement. Refinement of *F*^2^ against ALL reflections. The weighted *R*-factor *wR* and goodness of fit *S* are based on *F*^2^, conventional *R*-factors *R* are based on *F*, with *F* set to zero for negative *F*^2^. The threshold expression of *F*^2^ > σ(*F*^2^) is used only for calculating *R*-factors(gt) *etc*. and is not relevant to the choice of reflections for refinement. *R*-factors based on *F*^2^ are statistically about twice as large as those based on *F*, and *R*- factors based on ALL data will be even larger.

### Fractional atomic coordinates and isotropic or equivalent isotropic displacement parameters (Å^2^)

**Table:**

	*x*	*y*	*z*	*U*_iso_*/*U*_eq_	
Br1	0.12621 (8)	0.03733 (3)	0.83940 (3)	0.01337 (12)	
Cu1	0.5000	0.0000	1.0000	0.01824 (18)	
N1	0.5100 (7)	0.1368 (2)	1.0454 (2)	0.0154 (7)	
C2	0.6317 (9)	0.2051 (3)	0.9789 (3)	0.0158 (8)	
H2	0.7116	0.1869	0.9081	0.019*	
C3	0.6464 (9)	0.2998 (3)	1.0082 (3)	0.0153 (8)	
C4	0.5247 (9)	0.3257 (3)	1.1112 (3)	0.0165 (8)	
H4	0.5317	0.3899	1.1350	0.020*	
C5	0.3921 (9)	0.2560 (3)	1.1791 (3)	0.0163 (8)	
H5	0.3024	0.2725	1.2490	0.020*	
C6	0.3925 (8)	0.1636 (3)	1.1440 (3)	0.0160 (8)	
H6	0.3057	0.1165	1.1916	0.019*	
C7	0.7889 (9)	0.3717 (3)	0.9301 (3)	0.0211 (9)	
H7A	0.8971	0.3389	0.8690	0.032*	
H7B	0.9531	0.4110	0.9719	0.032*	
H7C	0.6094	0.4120	0.8986	0.032*	

### Atomic displacement parameters (Å^2^)

**Table:**

	*U*^11^	*U*^22^	*U*^33^	*U*^12^	*U*^13^	*U*^23^
Br1	0.0162 (2)	0.0089 (2)	0.01488 (19)	−0.00020 (14)	−0.00062 (13)	0.00010 (13)
Cu1	0.0286 (4)	0.0059 (3)	0.0194 (3)	0.0034 (3)	−0.0089 (3)	−0.0029 (2)
N1	0.0213 (16)	0.0084 (17)	0.0160 (15)	0.0025 (12)	−0.0051 (12)	−0.0015 (11)
C2	0.0178 (19)	0.015 (2)	0.0144 (17)	0.0028 (15)	−0.0028 (14)	−0.0033 (14)
C3	0.0138 (19)	0.012 (2)	0.0199 (19)	−0.0013 (14)	−0.0027 (14)	0.0015 (14)
C4	0.019 (2)	0.0076 (19)	0.0221 (19)	−0.0002 (15)	−0.0038 (15)	−0.0030 (14)
C5	0.0191 (18)	0.017 (2)	0.0132 (18)	0.0000 (15)	0.0024 (14)	−0.0019 (14)
C6	0.0156 (19)	0.013 (2)	0.0193 (19)	−0.0044 (14)	−0.0007 (15)	0.0010 (14)
C7	0.024 (2)	0.016 (2)	0.023 (2)	−0.0033 (16)	0.0020 (16)	0.0028 (15)

### Geometric parameters (Å, º)

**Table:**

Br1—Cu1	2.4351 (8)	C3—C7	1.506 (5)
Cu1—N1^i^	2.004 (3)	C4—C5	1.393 (5)
Cu1—N1	2.004 (3)	C4—H4	0.9500
Cu1—Br1^i^	2.4351 (8)	C5—C6	1.369 (5)
N1—C6	1.338 (4)	C5—H5	0.9500
N1—C2	1.351 (5)	C6—H6	0.9500
C2—C3	1.382 (5)	C7—H7A	0.9800
C2—H2	0.9500	C7—H7B	0.9800
C3—C4	1.388 (5)	C7—H7C	0.9800
			
N1^i^—Cu1—N1	180.000 (1)	C3—C4—C5	119.0 (4)
N1^i^—Cu1—Br1	89.57 (8)	C3—C4—H4	120.5
N1—Cu1—Br1	90.43 (8)	C5—C4—H4	120.5
N1^i^—Cu1—Br1^i^	90.43 (8)	C6—C5—C4	119.3 (3)
N1—Cu1—Br1^i^	89.57 (8)	C6—C5—H5	120.4
Br1—Cu1—Br1^i^	180.0	C4—C5—H5	120.4
C6—N1—C2	117.6 (3)	N1—C6—C5	122.8 (3)
C6—N1—Cu1	120.3 (3)	N1—C6—H6	118.6
C2—N1—Cu1	122.1 (2)	C5—C6—H6	118.6
N1—C2—C3	123.6 (3)	C3—C7—H7A	109.5
N1—C2—H2	118.2	C3—C7—H7B	109.5
C3—C2—H2	118.2	H7A—C7—H7B	109.5
C2—C3—C4	117.7 (3)	C3—C7—H7C	109.5
C2—C3—C7	120.6 (3)	H7A—C7—H7C	109.5
C4—C3—C7	121.8 (3)	H7B—C7—H7C	109.5
			
Br1—Cu1—N1—C6	117.1 (2)	N1—C2—C3—C7	179.4 (3)
Br1^i^—Cu1—N1—C6	−62.9 (2)	C2—C3—C4—C5	−0.6 (5)
Br1—Cu1—N1—C2	−62.5 (3)	C7—C3—C4—C5	179.2 (3)
Br1^i^—Cu1—N1—C2	117.5 (3)	C3—C4—C5—C6	1.6 (5)
C6—N1—C2—C3	1.2 (5)	C2—N1—C6—C5	−0.1 (5)
Cu1—N1—C2—C3	−179.1 (3)	Cu1—N1—C6—C5	−179.8 (3)
N1—C2—C3—C4	−0.9 (5)	C4—C5—C6—N1	−1.3 (5)

Symmetry code: (i) −*x*+1, −*y*, −*z*+2.

### Hydrogen-bond geometry (Å, º)

**Table:**

*D*—H···*A*	*D*—H	H···*A*	*D*···*A*	*D*—H···*A*
C2—H2···Br1^ii^	0.95	2.83	3.549 (4)	133
C6—H6···Br1^iii^	0.95	2.79	3.529 (4)	135
C5—H5···Br1^iv^	0.95	2.99	3.668 (4)	130

Symmetry codes: (ii) *x*+1, *y*, *z*; (iii) −*x*, −*y*, −*z*+2; (iv) *x*, −*y*+1/2, *z*+1/2.
